# Endophytic *Aspergillus fumigatiaffinis*: Novel paclitaxel production and optimization insights

**DOI:** 10.1007/s00253-024-13230-2

**Published:** 2024-07-03

**Authors:** Marwa Obiedallah, Alaa A. Yasien, Sabah S. Mohamed, M. Bassam Aboul-Nasr

**Affiliations:** Microbiology Lab., Botany and Microbiology Department, Faculty of Science, Sohag University, Sohag, 82524 Egypt

**Keywords:** *Aspergillus fumigatiaffinis*, Endophytic, Paclitaxel, Taxol®, Optimization, Design of experiment

## Abstract

**Abstract:**

This study investigated the potential of endophytic fungi to produce paclitaxel (Taxol®), a potent anticancer compound widely employed in chemotherapy. This research aimed to identify, confirm, and characterize endophytic fungi capable of paclitaxel (PTX) production and assess their paclitaxel yield. Additionally, it aimed to investigate factors influencing paclitaxel production. A total of 100 endophytic fungal isolates were collected and identified from the roots of *Artemisia judaica*. *Aspergillus fumigatiaffinis* exhibited the highest PTX production (26.373 μg L^−1^) among the isolated endophytic fungi. The strain was identified as *A*. *fumigatiaffinis* (Accession No. PP235788.1). Molecular identification confirmed its novelty, representing the first report of PTX production by *A*. *fumigatiaffinis*, an endophyte of *Artemisia judaica*. Optimization through full factorial design of experiments (DOE) and response surface methodology (RSM) significantly enhanced PTX production to 110.23 μg L^−1^ from 1 g of dry weight of the fungal culture under optimal conditions of pH 8.0, 150 μg L^−1^ becozyme supplementation, and 18 days of fermentation in potato dextrose broth. The presence of paclitaxel was confirmed using thin layer chromatography, high performance liquid chromatography, and gas chromatography–mass spectrometry. These findings maximize the role of endophytic fungus to produce a secondary metabolite that might be able to replace the chemically produced PTX and gives an opportunity to provide a sustainable source of PTX eco-friendly at high concentrations.

**Key points:**

*• Endophytic fungi, like A. fumigatiaffinis, show promise for eco-friendly paclitaxel production*

*• Optimization strategies boost paclitaxel yield significantly, reaching 110.23 μg L* ^*−1*^

*• Molecular identification confirms novelty, offering a sustainable PTX source*

## Introduction

Endophytic fungi are a class of microorganisms that can live within plant tissues in a mutualistic relationship without causing any harm to plants (Wang et al. 2016). These fungi are privileged by their ability to produce beneficial secondary metabolites with a wide range of biological functions (Dreyfuss and Chapela 1994). Therefore, endophytic fungi are being investigated by both the pharmaceutical and agricultural industries to discover active compounds can be produced by such fungi (Dreyfuss and Chapela 1994).

A large genus in the Asteraceae (Compositae) family is *Artemisia* (common name: Mugwort), which are aromatic herbs and shrubs. According to El-Sahhar et al. (2011), *Artemisia* plant is represented by four wild species (*Artemisia monosperma* Delile, *A*. *judaica* L., *A*. *scoparia* Waldst, and *A*. *verlotiorum* Lamotte), and one cultivated species (*A*. *vulgaris* L.) (Boulus 2002) in Egypt. In this study, the host *A*. *judaica* L. was selected for fungal isolation to detect the presence of fungal active bio compounds.

The tetracyclic diterpenoid Taxol®, sometimes referred to as paclitaxel (PTX), is found naturally in the Pacific evergreen tree (*Taxus brevifolia*), a member of the yew family *Taxaceae*. The molecular structure of PTX was first released in 1971 and then proceeded through clinical trials in 1984 (Wani et al. 1971). It is active against certain cancerous tumors including breast, lung, bladder, prostate, and head and neck cancer (Brown 2003). The extraction of PTX from *T*. *brevifolia* bark is an expensive and resource-intensive procedure that poses a significant risk to the environment. Additionally, the technique is largely circumvented by the possibility of extensive yew forest devastation (Malik et al. 2011). Furthermore, the cost for producing PTX from *T*. *brevifolia* was ten times higher than the funding allocated for the clinical trials (Yan-Hua et al. 2020). Consequently, New, dependable production routes—whether chemical, physical, or biological—were thus highly demanding. Numerous techniques have been developed during the last 20 years to produce PTX. Chemical processes have been used to create PTX, but the procedures need a lot of phases and steps, and the yield is low (Nicolaou et al. 1994). The PTX precursors have been semi-synthesized using the chemical modification method. However, it is an expensive and time-consuming procedure to extract these precursors, specifically baccatin III or 10-deacetylbaccatin III (Patel 1998). As a result, there is now a lot of interest in investigating PTX sources other than chemical method, namely fungal endophytes (Kusari et al. 2012). Therefore, the exploration of additional fungal isolates from a variety of natural environments could aid in the discovery of superior starter strains that exhibit a higher PTX productivity (Hao et al. 2013). *Taxomyces andreanae* was the first endophytic fungus to be described to produce PTX in 1993 (Stierle et al. 1993). Over twenty endophytic fungi have been documented to produce PTX globally, such as *T*. *Anderanae*, *Alternaria alternate*, and *Fusarium* sp. (Zhou et al. 2010). Currently, endophytic moulds are used as a more affordable and effective substitute method for producing and manufacturing PTX (Wang et al. 2000). Because endophytic fungi grow quickly, are inexpensive, robust against climatic change, can be grown on bulk fermenters, and are amenable to genetic modification, they have created a new avenue for the large-scale production of PTX (Stierle et al. 1993).

This study aims to isolate endophytic fungi associated with *Artemisia judaica*, screening the obtained isolates for their potentiality to produce PTX. Chemical confirmation of producing PTX and optimization of potent fungal isolates during culture conditions was obtained.

## Material and method

Paclitaxel (6 mg/mL) was purchased from Hikma-specialized pharmaceuticals, Egypt. Aluminum thin layer chromatography (TLC) plates were purchased from Sigma Aldrich, USA. Other chemicals were of analytical grade.

### Isolation of associated fungi with *Artemisia judaica’s* roots

Samples of healthy *Artemisia judaica* roots were collected from Wadi Abu Shih area region at Red Sea governorate (latitudes 26° 30′ and 26° 44′ N and longitudes 33° 20′ and 33° 30′ E). Three types of fungi were isolated: rhizosphere (i.e., fungi that closely surround plant roots and support their growth), rhizoplane (i.e., fungi interact with plant roots’ surface), and endophytic fungi (i.e., fungi that colonize within the plant tissue and exchanges mutualistic effect on each other). Two culturing media were used: Czapek’s agar (CZA) (glucose 20 g, NaNO_3_ 2.0 g, KH_2_PO_4_ 0.5 g, KCl 0.5 g, MgSO_4_·7H_2_O 2.0 g L^−1^; pH 8.5 ± 0.02) and its modified form Cellulose- Czapek’s agar (CCZA) which contains cellulose instead of glucose as the sole carbon source. First, rhizosphere fungi (RSF) were isolated by adding 5 g root sample into 100 mL sterile purified water and mixed thoroughly. The produced suspension (10 mL) was transferred into 40 mL. Finally, the plates were inoculated with 1 mL suspension. Second, *Artemisia* root was washed with sterile purified water and cut into small pieces for rhizoplane fungi (RPF) isolation, and 4 segments were placed per plate. Third, for endophytic fungi (EPF) isolation, *Artemisia* roots were first cut into small fragments (~ 5 × 5 mm) using sterile blade. Then, sterilized by successive soaking in 70% ethanol for 1 min and then 2.5% NaClO for 3 min, rinsed thrice with sterile purified water and dried using sterile paper tissues. All plates were incubated for 5 days at 28 ± 2.0 °C.

### Screening and extraction of paclitaxel produced by endophytic fungi

Isolated fungi were tested for their ability to produce paclitaxel (PTX) naturally by cultivation in 50 mL freshly prepared potato dextrose broth (PDB) medium (potato extract infusion of 200 g potatoes/L; prepared by boiling for 30 min and then filtrated through cheese cloth; 20 g/L dextrose was added at pH 6.5). Cultures were incubated at 28 ± 2.0 °C for 18 days statically. After incubation, mycelia were separated by filtration. For TLC detection, the mycelia were thoroughly crushed in a mortar, after that the fermented broth and ground mycelia were extracted by 50 mL chloroform. For HPLC and GC/MS analysis, 1 g of each culture was dried at 60 °C for 5 h and then immersed in chloroform for 24 h. Next day, chloroform was separated by filtration through anhydrous sodium sulfate. Finally, chloroform was evaporated to obtain the dried crude extracts. The crude extracts were redissolved in less amount of chloroform for the subsequent separation. Paclitaxel production was identified and detected from fungal crude extracts by spotting 50 μL on TLC plates comparing to authentic sample, silica gel coated with fluorescent indicator F254. The TLC plates were developed using chloroform: methanol (7:1, v/v) solvent system (Strobel et al. 1996). After the plates were air-dried, they were sprayed with a vanillin-H_2_SO_4_ reagent (2 g of vanillin dissolved in a mixture of 100 mL methanol and 1 mL H_2_SO_4_) and then incubated at 110 °C for 15 min. Also, the isolates showing intense bands of PTX on TLC were further confirmed using high-performance liquid chromatography (HPLC) analysis. The HPLC analysis (HPLC Chromass Young, Korea) was performed by Series equipped with a quaternary pump, a kinetex evo-C18 column 100 mm HPLC × 4.6 mm (Phenomenex®, USA), and operated at 35 °C. The injected volume was 20 μL. The UV detector was set at a wavelength of 205 nm, and the humidity was 38% rH.

### Molecular identification of *Aspergillus fumigatiaffinis*

A culture of the interested fungal isolate (AA17) was sent to Macrogen (Seoul, South Korea) for molecular identification and sequencing analysis using the universal primers: ITS1 (5′-TCCGTAGGTGAACCTGCGG-3′) and ITS4 (5′-TCCTCCGCTTATTGATATGC-3′). The obtained data files contained a consensus sequence of ITS1 and ITS4 primers, which was used to run a BLAST search on NCBI website for matching identification with deposited related species.

### Impact of various media types on paclitaxel production by *Aspergillus fumigatiaffinis* PP235788

A 7-day culture was used (1 mL spore suspension) to inoculate 49 mL medium of potato dextrose broth (PDB), Czapekʼs-Dox broth (CZB), M1D, and malt yeast extract broth (MYE). Each type of medium was inoculated in triplicates. Negative controls of each medium without inoculation were also considered. All conical flasks were incubated at 28 ± 2.0 °C for 2 weeks statically. Following incubation, microbial cultures were separated by filtration, and the previously described extraction and determination of PTX were carried out.

### Quantification of paclitaxel produced by *Aspergillus fumigatiaffinis* PP235788

*Aspergillus fumigatiaffinis* PP235788 extract was analyzed by GC/MS (unknown concentration of PTX) where the concentration was measured using a calibration curve of standard PTX. The calibration curve was performed by injecting five concentrations of standard PTX (30, 50, 80, 100, and 150 ppb ≈ μg L^−1^). An average of five independent trials was used to assess the concentration of PTX produced from extracted fungal filtrate. A TSQ triple quadrupole GC/MS instrument coupled with a Thermo Scientific™ TRACE™ 1300 GC (Thermo Scientific, Austin, TX, USA) was used. Sample’s introduction was performed by Thermo Scientific™ AS3000 autosampler and chromatographic separation using a Thermo Scientific™ TraceGOLD TG-5MS 30 m × 0.25 mm I.D. × 0.25-μm-film capillary column.

### Optimizing paclitaxel production by *Aspergillus fumigatiaffinis* PP235788

Based on the impact of various types of media on PTX production, potato dextrose broth medium was selected for performing optimization conditions in addition to adding a stimulator for PTX production, which is vitamin B complex (becozyme) at different concentrations per liter. Becozyme is a vitamin B complex which includes thiamine hydrochloride (B_1_, 10 mg), riboflavin sodium phosphate (B_2_, 4.57 mg), nicotinamide (B_3_, 40 mg), dexpanizole (B_5_, 6 mg), and pyridoxine hydrochloride (B_6_, 6 mg). Also, pH values and days of incubation were considered.

The design of experiment (DOE) statistical analysis was employed to assess the individual and interactive effects of 3 variables on PTX production using SigmaXL Version 10 (SigmaXL Inc., Ontario, Canada) (Jankovic et al. 2021). An experimental matrix of 2-level factorial design (8-run, full factorial) was designed upon 3 variables (becozyme, pH value, and incubation days). Each factor is represented by 2-levels of “low” and “high” to measure one response (PTX production μg L^−1^). The settings were “50, 150 μg L^−1^,” “5.0, 8.0,” and “14, 18” for becozyme, pH, and fermentation days, respectively (Table 1). Two biological replicates were used to maximize standard deviation value. The order of experimental runs was randomized to minimize the impact of any uncontrolled variables. All runs were performed in 250-mL-Erlenmeyer flasks with 50 mL PDB at 28 ± 2.0 °C with freshly prepared fungal spore suspension (1 mL). After growth, PTX was extracted and quantified by TLC and HPLC, as described earlier.

**Table 1:** Design of 3 factor, 8-run, 2^**^3, full-factorial for one response (paclitaxel production μg L^−1^). One block and one center point

**Run order**	**Std. order**	**A: becozyme**	**B: pH value**	**C: incubation days**	**Paclitaxel conc.**	**Predicted (fitted) values**	**Residuals**
1	5	50	5	18	4.98	5.325	−0.345
2	12	150	8	14	70.14	69.635	0.505
3	3	50	8	14	30.46	30.065	0.395
4^*^	16	**150**	**8**	**18**	**110.23**	109.87	0.365
5	2	150	5	14	20.11	19.785	0.325
6	4	150	8	14	69.13	69.635	−0.505
7	10	150	5	14	19.46	19.785	−0.325
8^*^	8	**150**	**8**	**18**	**109.5**	109.87	−0.365
9	15	50	8	18	50.11	49.940	0.170
10	1	50	5	14	3.25	3.045	0.205
11	9	50	5	14	2.84	3.045	−0.205
12	11	50	8	14	29.67	30.065	−0.395
13	14	150	5	18	11.16	10.645	0.515
14	7	50	8	18	49.77	49.940	−0.170
15	13	50	5	18	5.67	5.325	0.345
16	6	150	5	18	10.13	10.645	−0.515

* Runs 4 & 8 indicate the highest production of PTX under optimized conditions

After determining the optimum conditions for PTX production among the 3 desired variables, it was found that pH and becozyme are the most important factors for production. Thus, these two factors were selected for the response surface design (RSD) analysis. Response surface approach is a very useful tool for determining the best circumstances to boost microbial production and for building correlational mathematical models which predict the response variable based on different parameter combinations. Central composite design (CCD, 2 ctr pts) was employed using 2 replicates, and alpha axial value was set to face centered (*alpha* = 1.0) for 10-run. The used design is depicted in Table 2; each factor is represented by 3 center points (low, medium, and high).

**Table 2:** Response surface methodology of PTX production. Paclitaxel production, predicted values, and residuals using 2 variables through 20-run

**Run order**	**Std. order**	**Center points**	**A: becozyme**	**B: pH**	**Paclitaxel conc.**	**Predicted (fitted) values**	**Residuals**
1	8	1	150	11	73.4	74.594	−1.194
2	11	1	50	5	7.23	7.690	−0.4510
3	16	1	250	8	46.35	46.722	−0.372
4	10	0	150	8	110.5	109.89	0.607
5	2	1	250	5	8.77	8.725	0.045
6	1	1	50	5	7.44	7.690	−0.249
7	7	1	150	5	74.01	73.080	0.929
8	14	1	250	11	13.11	12.608	0.501
9	4	1	250	11	13.13	12.608	0.521
10	19	0	150	8	109.7	109.89	−0.192
11	5	1	50	8	44.01	43.317	0.692
12	17	1	150	5	73.99	73.080	0.909
13	18	1	150	11	73.32	74.594	−1.274
14	12	1	250	5	7.55	8.725	−1.175
15	15	1	50	8	41.89	43.317	−1.427
16	3	1	50	11	7.12	6.833	0.286
17	13	1	50	11	7.99	6.833	1.157
18	20	0	150	8	109.7	109.89	−0.192
19	9	0	150	8	110.3	109.89	0.407
20	6	1	250	8	47.2	46.722	0.477

The two generated models, full-factorial design, and response surface methodology were statistically analyzed using two-way ANOVA, for detecting the significance of two variables, pH value, and becozyme concentration on produced concentration of PTX.

### Statistical analyses

All experiments were conducted with two or three biological replicates, as indicated, and the results were expressed by *mean* ± *standard deviation*. Statistical analyses and analysis of variance (ANOVA) for each model is generated using SigmaXL Version 10.

### Deposition of fungal material

The isolate *A*. *fumigatiaffinis* AA17 was deposited at the Assiut University Mycological Centre (AUMC), Egypt, with deposit number AUMC16334 and accession number PP235788.1 at GenBank.

## Results

### Isolation and screening of endophytic fungi for paclitaxel production

**Fig. 1:**
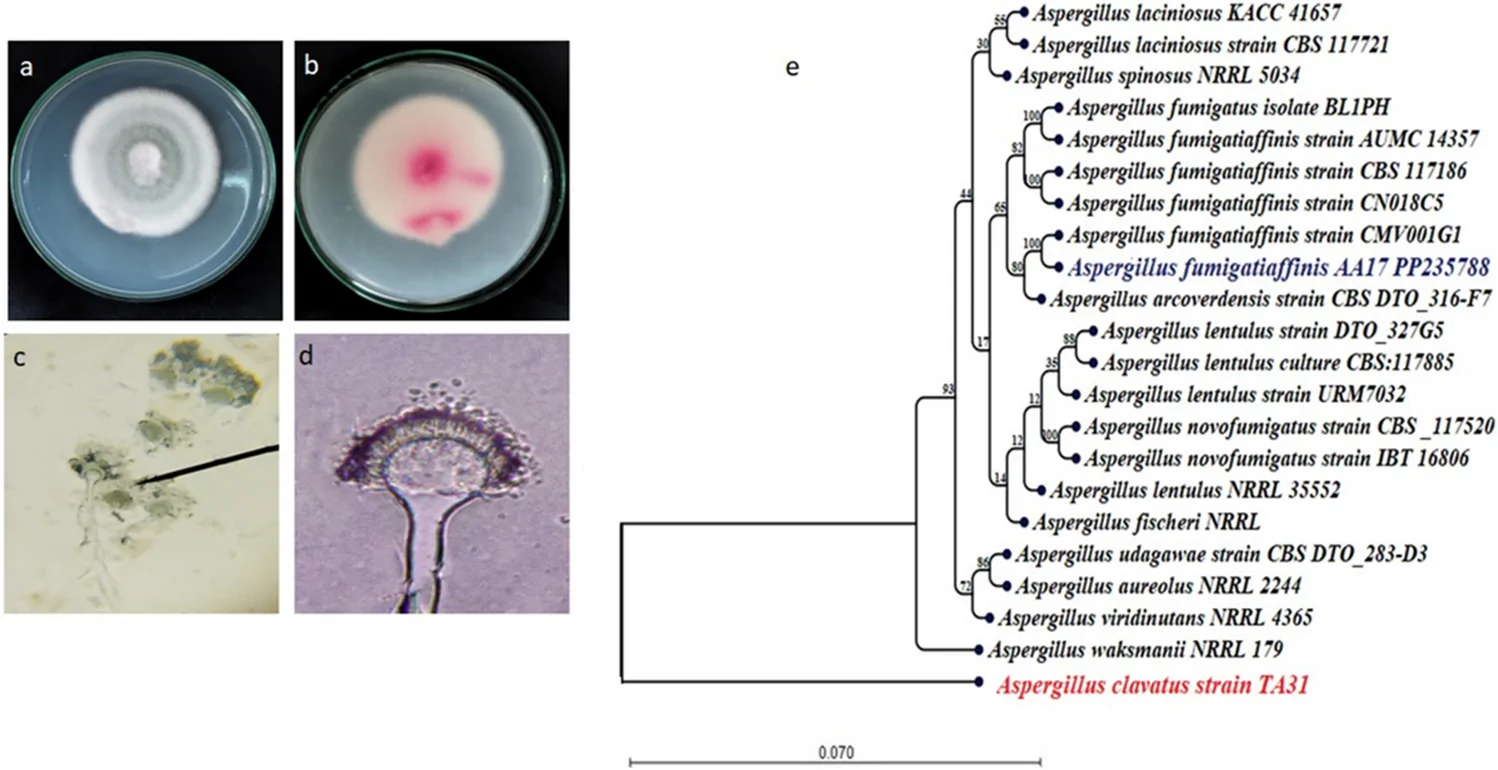
Morphological characterization of *A. fumigatiaffinis*. **a**, **b** Colony and reverse color on CCZA medium, **c**, **d** conidial head and conidia under light Microscope at 10× and 40×, respectively. **e** Maximum parsimony phylogenetic tree generated from ML/MP analysis using a heurestic search of 1000 replications based on ITS sequence data of *Aspergillus fumigatiaffinis* strains AA17 (in blue) compared to the most similar species’ sequences belonging to *Aspergillus*: section *Fumigati*. Bootstrap support values for ML/MP ≥ 50% are indicated at the respective nodes. The tree is rooted to *Aspergillus clavatus* strain TA 31 as out group (in red)

A total of 280 fungal isolates associated with *Artemisia judaica*’s root were isolated (RSF, RPf, and EPF). *Aspergillus* was the most dominant genus (200 out of 280). Among all isolated fungi, the most frequent species was *A. fumigatiaffini* which was morphologically identified according to Samson et al. (2007) . It exhibited a velvet white colony color interspersed with gray-green patches of conidia, with a pink reverse on CCZA and PDA (Fig. 1). Figure 1 shows the conidial heads are short, columnar, and uniseriate; conidiophores are smooth walled; vesicles are usually globose to sub-globose in shape; and conidia are smooth globose to broadly ellipsoidal (2–3 μm in diameter). Thus, *Aspergillus fumigatiaffinis* AA17 was molecularly identified by analyzing the nucleotide sequence of the amplified 18S rRNA gene. Through BLAST search tool on NCBI database, it was found that AA-17 is 100.00% similar to *A*. *fumigatiaffinis* strain CMV001G1 (MK450913.1), with zero *E*. values, and 100.00% query coverage. After that, sequences of 21 most similar species in *Aspergillus* section*: Fumigati* were downloaded from GenBank database. Twenty-two sequences were aligned; terminals were eliminated; and phylogenetic tree (Fig. 1) was constructed using UPGMA statistical method at 1000 bootstrap replications using CLC genomics workbench (version 24.0). *Aspergillus clavatus strain* TA31 (HQ392483.1) was used as an outgroup in the phylogenetic tree.

**Fig. 2:**
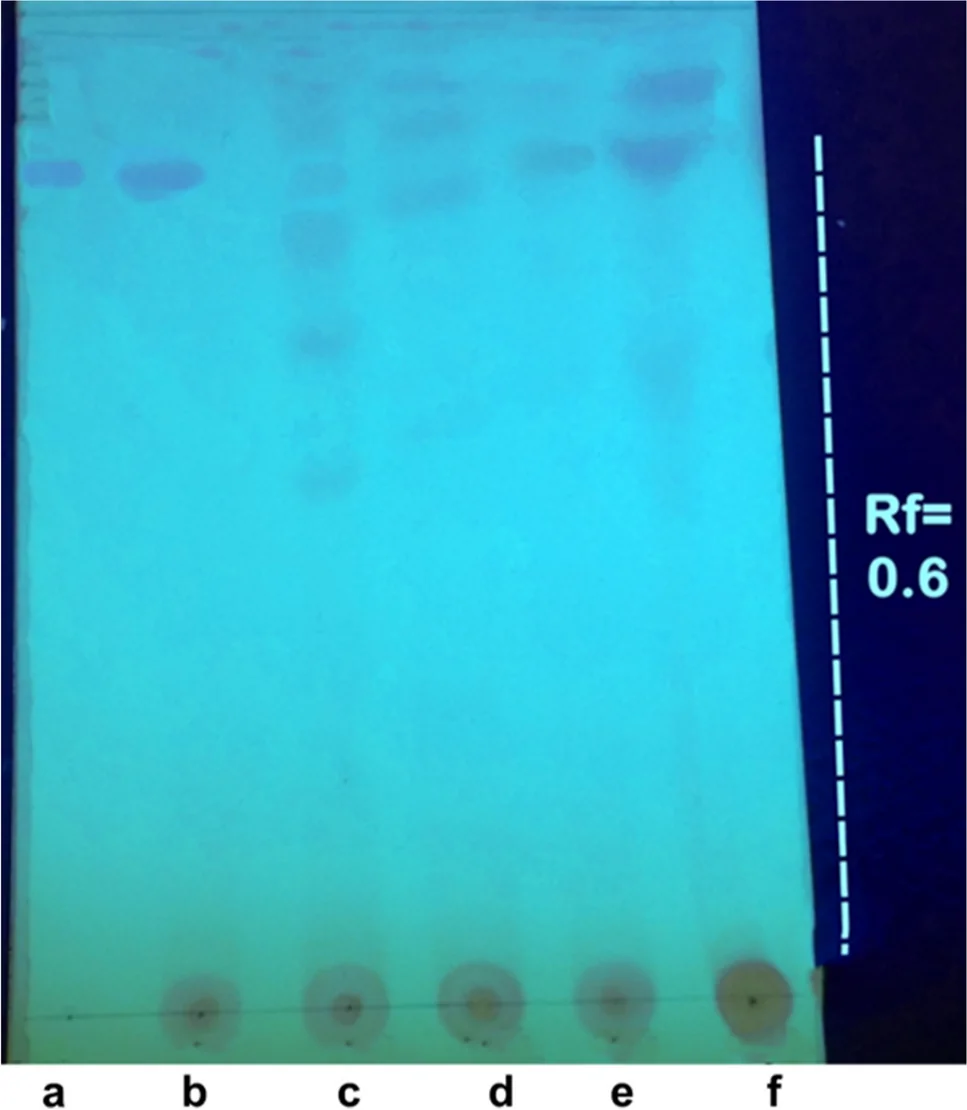
Chromatographic analysis of the extracted PTX using TLC, visualization at 254 nm. **a** Standard paclitaxel, **b** the extract of fungal isolate AA17 after optimization, **c** the extract of fungal isolate AA17 before optimization on M1D, **d** Czapek-Dox’s, **e** malt yeast extract, and **f** potato dextrose broth media

**Fig. 3:**
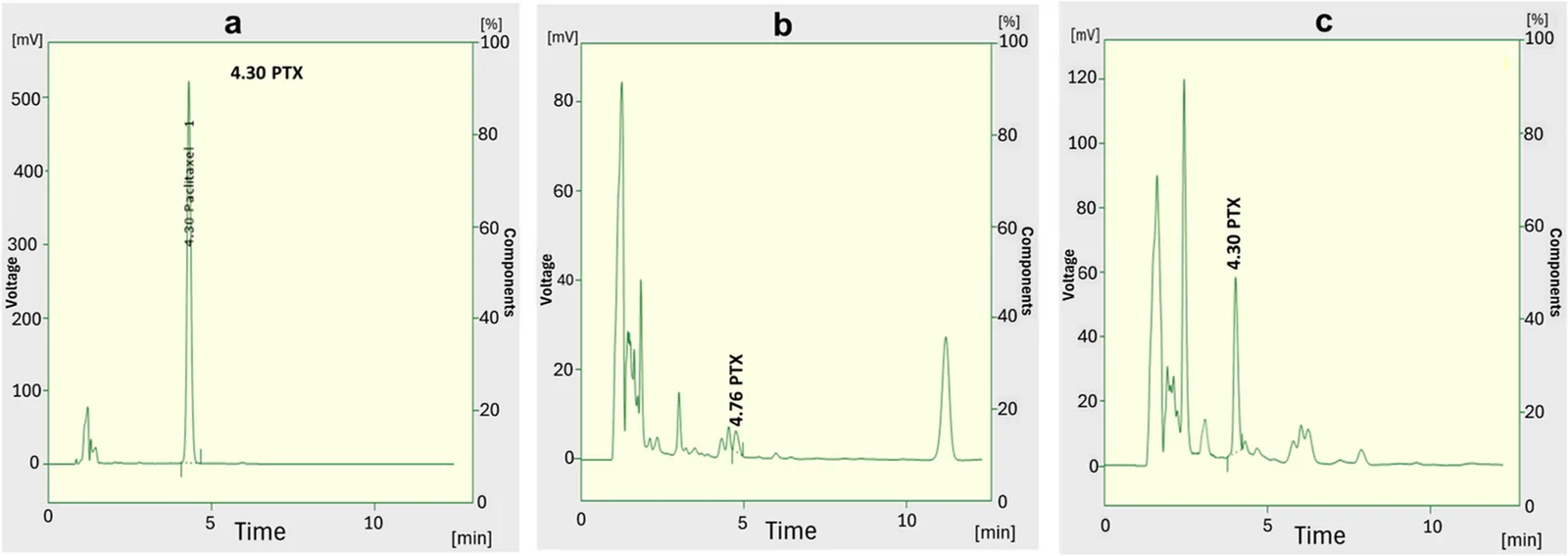
High-performance liquid chromatography (HPLC) chromatograms. **a** Standard PTX, **b** extract of *Aspergillus fumigatiaffinis* under normal conditions, **c** extract of *Aspergillus fumigatiaffinis* after optimization by adding 150 μg L^−1^ bicozyme at pH 8.0 after 18 days incubation

Through screening isolated endophytic fungal strains (EPF) for PTX production, it was found that 60 out of 100 isolates showed positive results for PTX production: 40 of *A. fumigatiaffinis* and 20 of *Aspergillus fischeri* isolates. The fungal extracts were evaluated for the presence of PTX through TLC analysis. The TLC plates were visualized at 254 nm (short wavelength). The *R*_*f*_ values of PTX bands along with the authentic sample was 0.6 (Fig. 2) and exhibited a bluish spot that turned into a dark gray color upon spraying with 1% vanillin (w/v) in sulfuric acid under mild heat. Paclitaxel bands of *Aspergillus fumigatiaffinis* extracts on TLC sheet were more intense than other tested fungi peaks. The maximum PTX productivity was achieved by *Aspergillus fumigatiaffinis* AA17 (26.373 μg L^−1^) after TLC and HPLC measurements at retention time 26.40 min (Fig. 3).

### Influence of medium composition on paclitaxel production, and quantification using *A. fumigatiaffinis* PP235788

**Fig. 4:**
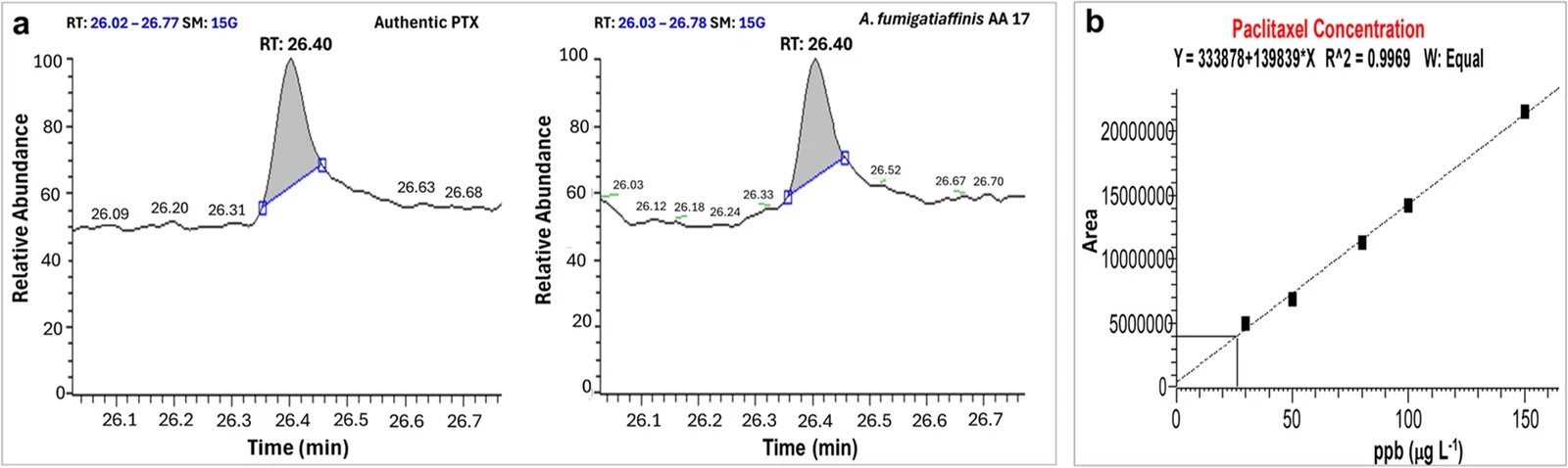
Gas chromatography/mass spectrometry (GC/MS) of paclitaxel. **a** Charts of authentic PTX and fungal PTX produced by the isolate AA17 on PDB at 26.40 min, **b** calibration curve using 5 concentrations of authentic PTX

By cultivating on PDB, CZB, M1D, and MYE, the impact of medium composition on the generation of PTX by *A*. *fumigatiaffinis* PP235788 has been assessed. Based on the findings presented in Fig. 2, it can be inferred that *A*. *fumigatiaffinis* PP235788 produced the highest amount of PTX when grown on PDB, followed by MYE. Both showed distinct corresponding PTX spots on the TLC plates. The produced PTX on PDA medium was quantified using GC/MS (Fig. 4). Using the equation (*Y* = 333878 + 139839 × *X*), linear calibration curves for PTX samples were obtained with correlation factors more than 0.9969. The most intense band of PTX on TLC plate produced from PDB, followed by MYE, then M1D and the weakest band produced from CZB (Fig. 2), which confirms that possibility to use this fungus for PTX production using PDB medium. Therefore, additional optimization investigation has been conducted using the response surface methodology statistical approach to maximize the PTX yield by *A*. *fumigatiaffinis* PP235788.

### Optimized parameters improving PTX yield by *A. fumigatiaffinis* PP235788

#### Full factorial design of experiment

**Fig. 5:**
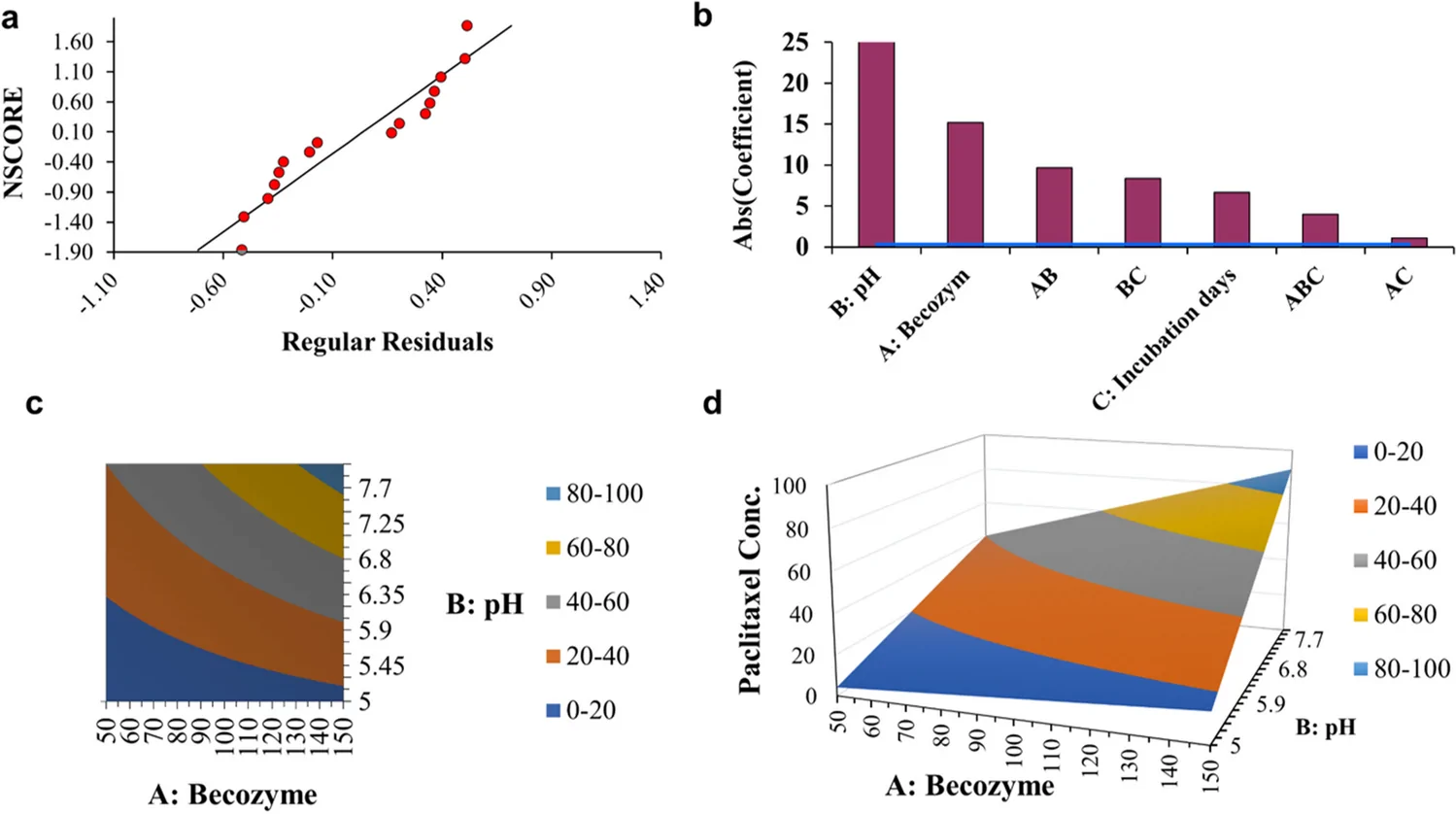
Plots of DOE statistical analysis for PTX production. **a** Normal probability plot of regular residuals of PTX production, **b** Pareto chart of coefficients, **c** DOE contour plot, and **d** DOE 3D surface plot

The design of experiment (DOE) multiple regression model was resolved using the equation: paclitaxel concentration (μg L^−1^) = (37.288125) + (15.194375) × A: becozyme + (27.588125) × B: pH + (6.655625) × C: incubation days + (9.679375) × AB + (1.116875) × AC + (8.370625) × BC + (3.971875) × ABC. The TLC and HPLC were used to determine the concentration of produced PTX. Experimental PTX production, predicted values, and residuals are all displayed in Table 1. At run #4, the highest expected (109.87 μg/mL) and experimental (110.23 μg mL^−1^) yield of PTX by *A*. *fumigatiaffinis* PP235788 using a full-factorial design was achieved by adding 150 μg L^−1^ becozyme as PTX stimulator to PDB medium, for 18 days of incubation, at initial pH 8.0, and 28 ± 2.0 °C statically. At this cultural run, the produced PTX was significantly increased by about four folds, compared to unoptimized conditions (26.373 μg L^−1^). The Pareto chart in Fig. 5 illustrates the priority of significance of the variables involved in the production of PTX, based on the effects of individual factors as shown by the full-factorial design, showing that pH value plays a crucial role in the PTX production, followed by the becozyme supplementation. The model R-square showed 99.99% and significant *P* value (< 0.05) for constant, the three factors, and the combination of factors (Table 3). The standard deviation of experimental error is 0.525613. The Pareto chart shows that pH value is the most important (*X*) factor affecting PTX production and that all the interactions (AB, BC, AC, and ABC) are also significant (*P* value = 0.0000) (Fig. 5).

**Table 3:** Analysis of variance (ANOVA) report of the full-factorial design model for PTX production by *A*. *fummigatiaffinis*

**Term**	**Coefficient**	**SE coefficient**^**a**^	***T***^**b**^	**VIF**^**c**^	**Tolerance**
**Parameter estimates (coded units)**					
Constant	37.288^***^	0.131	283.77	1	1
A: becozyme	15.194^***^		115.63		
B: pH	27.588^***^		209.95		
C: incubation days	6.656^***^		50.650		
AB	9.679^***^		73.662		
AC	1.117^***^		8.500		
BC	8.371^***^		63.702		
ABC	3.972^***^		30.227		
**Model summary**					
R-square	99.99%				
R-square adjusted	99.98%				
S (root mean square error)	0.525613				
***denote significance level of 1%, *P* = 0.0000 implies high significance					
^a^ Standard error of the coefficient					
^b^ *T*-value of a *T*-test performed on the coefficients					
^c^ Variance inflation factor					
Source	***DF***^**a**^	***SS***^**b**^	***MS***^**c**^	***F***^**d**^	***P***
**Analysis of variance for model**					
Model	7	19473	2781.8	10069	0.0000
Error	8	2.210	0.276		
Pure error	8	2.210	0.276		
Total (model + error)	15	19475	1298.3		

^a^Degrees of freedom

^b^Sum of squares

^c^Mean square,

^d^*F*-statistic (the ratio of the model mean square to the error mean square)

The contour and surface plots (Fig. 5) are generated with optimum center points of 100 μg L^−1^, 6.5, and 16 for becozyme concentration, pH value, and fermentation days, respectively. While after using the Data Excel solver to reach the lowest cost optimized experimental settings, we obtained 147.4 μg L^−1^, 7.8, and 17.9 for the three factors, with predicted response of 99.99.

#### The central composite design of response surface methodology

**Fig. 6:**
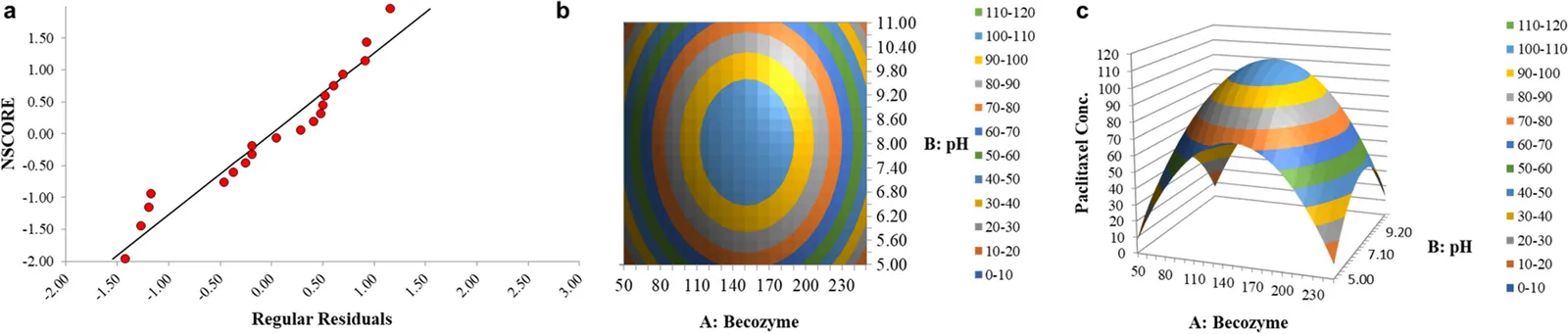
Response surface methodology of PTX production. **a** Normal probability plot of regular residuals, **b** contour plot, and **c** 3D surface plot

After discovering that pH value and becozyme concentration are the most important factors impacting PTX production, further optimization was conducted using the response surface methodology (RSM); the design is depicted in Table 2 using two biological replicates. The central composite design (CCD) analysis showed a center point at pH 8.0 and becozyme 150 μg L^−1^ (Fig. 6), where the highest concentration was achieved. The RSM regression model equation is paclitaxel conc. = (109.8928571) + (1.7025) × A: becozyme + (0.756666667) × B: pH + (1.185) × AB + (−64.87321429) × AA + (−36.05571429) × BB. Hence, the plots were generated (Fig. 6). Normal probability plot analysis demonstrates how “normal” the predictions were over the dependent variable’s value range (Fig 6). Table 4 shows the ANOVA report of the RSM model.

**Table 4:** Analysis of variance (ANOVA) report of the response surface methodology (*RSM*) for PTX production by *A*. *fummigatiaffinis*

Term	Coefficient	SE coefficient^**a**^	*T*^**b**^	***P***	VIF^**c**^	Tolerance
**Parameter estimates (coded units)**						
Constant	109.892	0.388	282.87	0.0000		
A: becozyme	1.7025	0.265	6.415	0.0000	1	1
B: pH	0.757	0.265	2.851	0.0128	1	1
AB	1.185	0.325	3.646	0.0026	1	1
AA	−64.873	0.426	−152.44	0.0000	1.029	0.972
BB	−36.055	0.426	−84.724	0.0000	1.029	0.972
**Model summary**						
R-square	99.96%					
R-square adjusted	99.95%					
S (root mean square error)	0.919					
^a^ Standard error of the coefficient						
^b^ *T*-value of a *T*-test performed on the coefficients						
^c^ Variance inflation factor						
Source	*DF*^**a**^	*SS*^**b**^	*MS*^**c**^	*F*^**d**^	***P***	
**Analysis of variance for model**						
Model	5	30236	6047.3	7155.1	0.0000	
Error	14	11.832	0.845			
Lack of fit	3	7.566	2.522	6.502	0.0086	
Pure error	11	4.267	0.388			

^a^Degrees of freedom

^b^Sum of squares

^c^Mean square

^d^*F*-statistic (the ratio of the model mean square to the error mean square)

## Discussion

Since fungal endophytes can grow quickly, ferment efficiently, are tolerant to variations in climate, and can be genetically modified, they have raised expectations for the industrial production of paclitaxel. However, the reduced repeatable yield and decreased productivity of PTX with sub-culturing of fungi raised doubt on their potential for industrial PTX production (Staniek et al. 2009; El-Sayed et al. 2019a; Abdel-Fatah et al. 2021; Abdel-Fatah et al. 2022). Most endophytic PTX-producing fungi were recovered from *Podocarpus* sp. and *Taxus* sp., both of which are members of the Taxaceae family (El-Sayed et al. 2019a; El-Sayed et al. 2021). A main objective in biotechnology field is to investigate the paclitaxel-producing endophytic fungus from plants outside the Taxaceae family that have potential Taxol productivity. Many reports confirmed that *Artemisia* genus contains active biological substances in abundance, including coumarin, flavonoids, sesquiterpenoids, and terpenoids (Tan et al. 1998; Bora and Sharma 2011). It was reported that PTX could be produced by endophytic fungi that have been isolated from various non-*Taxus* host 302 plants (Tan and Zou 2001; Kumala et al. 2007; ChangTian et al. 2008; Gangadevi and Balcanica 2008; Gangadevi and Muthumary 2009; Kumaran et al. 2009). One of the most widely used medicinal plants, *Artmisia judaica* is known for its pharmacological properties, such as: gastrointestinal disorders, insecticidal, antifeedant and antifungal activities (Liu et al. 2004; Abdelgaleil et al. 2008). Therefore, the aim of this investigation was to identify the endophytic fungal species from the *Artmisia judaica* other than the known plants that produce paclitaxel; and then, quantify, confirm, and optimize its production. Based on TLC examination, the strain *A*. *fumigatiaffinis* AA17 showed the greatest PTX yield (26.373 μg/L) among the screened recovered endophytic fungi. Hao et al. (2013) reported *Aspergillus* species such as *A*. *fumigatus*, *A*. *niger*, and *A*. *candidus* as producers of PTX, whereas our study marks the first documentation of PTX production by *A*. *fumigatiaffinis*. Also, to our knowledge this is the first report of isolating *A*. *fumigatiaffinis* from *Artmisia judaica* medicinal plant. The PTX concentration we obtained agreed with the concentrations reported from *Fusarium mairei* UH23 (20.0 μg/L), endophyte of *Taxus* × *media* (Dai and Tao 2008), and *Mucor rouxianus* DA10, *Taxus chinensis* endophyte which produced 30.0 of Taxol (Miao et al. 2009). Endophytes of many *Taxus* species, including, *A*. *niger* (Zhao et al. 2009), and *Fusarium solani* (Deng et al. 2009) were reported for production 273.46 and 163.35 μg/L, respectively. The highest Taxol production was reported in 2019 using *A*. *fumigatus* KU-837249 (1.60 g/L) (Kumar et al. 2019). In the current study, after optimizing culture conditions of *A*. *fumigatiaffinis*, we could reach a four-fold increase in production reaching 110.23 μg L^−1^, which presents a novel endophytic fungal strain of promising high paclitaxel yield. Repeating culturing this strain successively for biological replicates indicates its genetic stability for long-run utilization in paclitaxel production. The optimum conditions were culturing in PDB medium supplemented with 150 μg L^−1^ becozyme, pH8.0, and fermented for 18 days. Our results are consistent with Somjaipeng et al. (2015), El-Sayed et al. (2018), and El-Sayed et al. (2019d) who confirmed the effectiveness of PDB in affecting PTX productivity. Also, Staniek et al. (2009), Li et al. (2015), Somjaipeng et al. (2015), and El-Sayed et al. (2019c) reported that after 18 days of fermentation, the effectiveness of PTX production increases. Somjaipeng et al. (2015), El-Sayed et al. (2018), and Rui et al. (2011) also reported that pH 8.0 is favorable for PTX production. Vitamin B compounds (becozyme) addition to the media improves PTX productivity, as indicated by El-Sayed et al. (2019b), who demonstrated an increase in PTX concentration by *Aspergillus terreus*, where they could obtain 343.4 μg L^−1^ PTX after adding vitamin B supplements to the media. Yuan et al. (2006) indicated that when adding 50 mg of vitamin B to media, it stimulates the production of PTX.

In conclusion, this study unveils *Artemisia judaica* as a novel reservoir for endophytic fungi capable of producing paclitaxel, with *A*. *fumigatiaffinis* AA17 emerging as a promising strain. Through rigorous screening and optimization processes, we identified and characterized *A*. *fumigatiaffinis* PP235788 as a high-yielding producer of paclitaxel. Our results highlight the importance of employing advanced statistical analysis techniques, such as full factorial design of experiment and response surface methodology, to optimize paclitaxel production parameters effectively. Furthermore, our findings confirm the presence of paclitaxel in the fungal extract using various analytical techniques, thus validating its potential pharmaceutical relevance. Overall, this study contributes to the exploration of sustainable and cost-effective alternatives for paclitaxel production, offering promising prospects for cancer treatment and pharmaceutical development. Further research on genetic stability and scale-up production is warranted to realize the full potential of *A*. *fumigatiaffinis* in paclitaxel production.

## Data Availability

The original data presented in the current study are included in the article; further inquiries can be directed to the corresponding author.
